# The Expanded Vermiculite Was Quickly Prepared by the Catalytic Action of Manganese Dioxide on Hydrogen Peroxide and Its Adsorption Properties to Cd

**DOI:** 10.3390/molecules28020817

**Published:** 2023-01-13

**Authors:** Yunzhu Chen, Hongjuan Sun, Tongjiang Peng, Tongxi Gao, Wenjin Ding, Tao Hui, Lei Jiang

**Affiliations:** 1 Key Laboratory of Ministry of Education for Solid Waste Treatment and Resource Recycle, Southwest University of Science and Technology, Mianyang 621010, China; 2 Department of Resources & Environment, Xichang University, Xichang 615000, China; 3 Institute of Mineral Materials and Application, Southwest University of Science and Technology, Mianyang 621010, China

**Keywords:** vermiculite, exfoliation, hydrogen peroxide, manganese dioxide, catalysis

## Abstract

The structure and activity of vermiculite can be maintained by expanding vermiculite (Vrm) with hydrogen peroxide. However, it is time-consuming. In past studies, little attention has been paid to the catalytic properties of manganese dioxide on hydrogen peroxide to improve the swelling efficiency of vermiculite. In this experiment, this catalytic effect was utilized to swell Vrm in a short time. The samples were then used to adsorb Cd from the solution. Through a series of characterization tests. The results showed that the exothermic rate was 1960.42–2089.164 J/min and the total exothermic heat was 39,208.4–41,783.28 J when expanding 10 gVrm, which could have a good expansion effect. The expansion was completed in about 40 min. Compared with Vrm, the adsorption of Cd is enhanced by about 30%. It is consistent with the proposed secondary kinetic adsorption model. This study provides a new perspective and theoretical guidance for improving the efficiency of Vrm stripping by hydrogen peroxide. A kind of expanded Vrm with better Cd adsorption efficiency was also prepared.

## 1. Introduction

Vermiculite (Vrm) has excellent properties such as light weight, low thermal conductivity, thermal insulation, sound insulation, and adsorption, so it is widely used in construction, agriculture, thermal insulation, and environmental protection [1,2,3]. Vrm layers are mainly connected by hydrogen bonds and van der Waals forces [4]. If these forces are disrupted, Vrm will swell or even flake off [5]. The exfoliated Vrm sheets have a larger surface area [6], more reactive sites, and better electrical conductivity than the swollen Vrm [7]. These properties are beneficial for the adsorption of pollutants in the environment. Therefore, many scholars have studied the use of expanded Vrm to treat the environment [8,9,10,11,12].

In order to achieve Vrm expansion, methods such as high-temperature heating, microwave irradiation, and chemical immersion are usually used. The principle of high-temperature expansion is that water molecules in the interlayer domain of Vrm are heated and rapidly vaporized to form water vapor. As water vapor cannot be discharged in time, a strong pressure is formed between the layers. It causes the crystal layer to be propped up and the interlayer spacing to increase, resulting in Vrm expansion [13]. It has been demonstrated that high-temperature heating causes irreversible damage to the interlayer structure of Vrm, resulting in reduced mechanical strength and destruction of surface functional groups. Microwave irradiation has a similar mechanism to high-temperature heating [14]. However, microwaves do not destroy the lamellar structure of Vrm and maintain the structural integrity of Vrm [13,15]. At the same time, microwave is a green, clean, and efficient expansion method [16]. However, microwave expansion suffers from the problem of inhomogeneity [17]. In addition, it has an effect on the -OH on the surface of Vrm. Hydrogen peroxide is a common reagent for chemical expansion. This method can effectively protect the -OH functional group and even increase its content. It is important for the use of Vrm surface functional groups to adsorb cationic pollutants from the environment [18,19]. However, the biggest problem of this method is the long time consumption, which often exceeds 5 h [20] and even reaches 30 h [19].

In this paper, the chemical method of expansion Vrm is optimized. The main purpose is to use the catalytic properties of manganese dioxide for hydrogen peroxide. This catalytic reaction has received little attention in past studies. It is well known that manganese dioxide is a cheap and easily available inorganic reagent. It can be added only a little for catalysis and can be used repeatedly. It is also stable in the environment and does not cause secondary contamination [21].

This study first explored the amount of manganese dioxide addition. Then the solid–liquid ratio of Vrm and hydrogen peroxide was investigated. The appropriate addition amount can shorten the dissolution time and improve the efficiency. A reasonable solid–liquid ratio can reduce the waste of resources. After determining the addition amount and solid–liquid ratio, the prepared samples were also used to adsorb Cd from the solution. The theoretical basis was provided for the utilization of the samples in environmental treatment.

## 2. Results and Discussion

### 2.1. Effect of Manganese Dioxide on Expansion Properties

**Figure 1:**
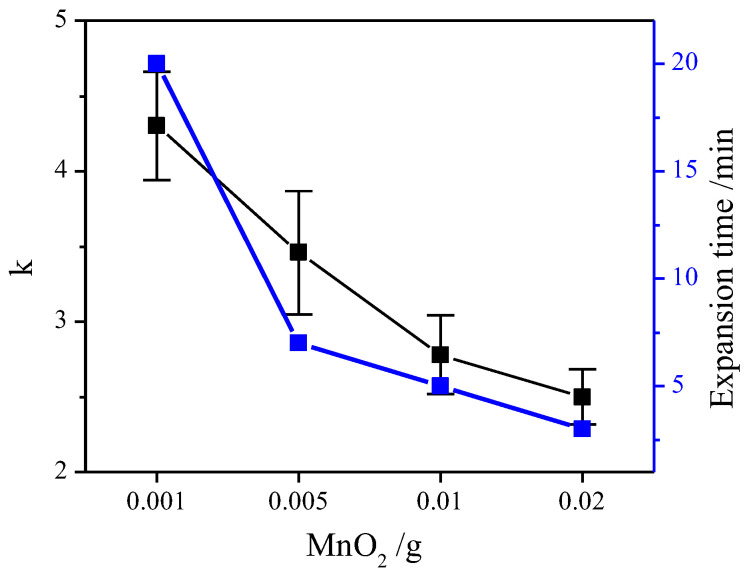
Effect of manganese dioxide on expansion rate and expansion time.

**Figure 2:**
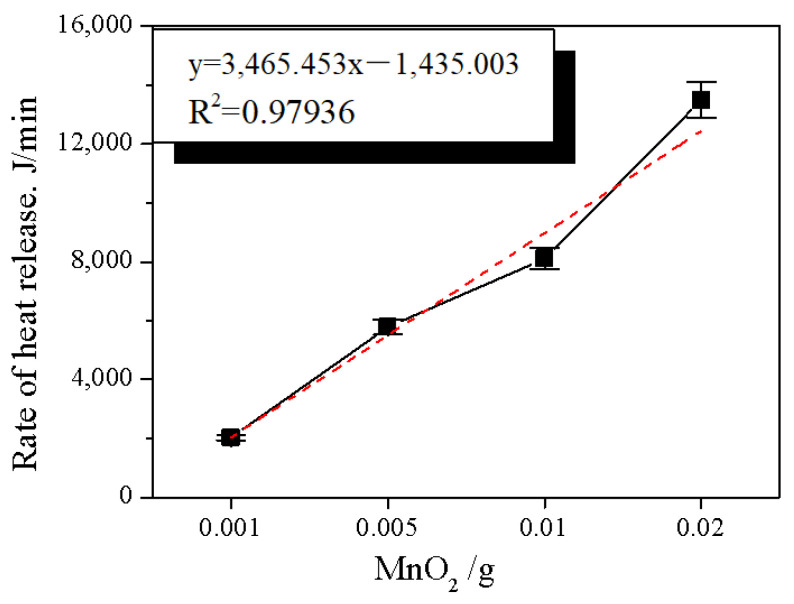
Effect of manganese dioxide on the exothermic rate.

In this experiment, the effect of manganese dioxide on the expansion time and expansion rate k was investigated just as shown in Figure 1. The result shows that the expansion time was significantly reduced with the increase in manganese dioxide. When the addition amount was 0.001 g, the reaction ended in about 20 min. When the addition amount was 0.02, the reaction ended in about 3 min. Meanwhile, the trend of k was the same as the expansion time. At the addition amount of 0.001 g, k ≈ 4.2. It was the largest. More than 50% increased over 0.02 g. It may be caused by the different amount of manganese dioxide addition. This result was further analyzed. The different addition amounts resulted in the change of exothermic rate. It was shown that when H_2_O_2_ decomposed at a concentration of 30%, the heat released was 670~714 J/g [22]. Since the 30% hydrogen peroxide used in each group was 40 mL, the total heat released by hydrogen peroxide was calculated to be 39,208.4~41,783.28 J. The relationship between the amount of manganese dioxide and the exothermic rate was then fitted linearly (Figure 2). The relationship was found to fit the univariate regression equation, R^2^ = 0.97567. The fit was good. The exothermic rate of 0.02 was more than 5 times that of 0.001. Therefore, it can be inferred that the addition of manganese dioxide was directly proportional to the exothermic rate of hydrogen peroxide. Meanwhile, the slower the exothermic rate, the higher the vermiculite expansion rate.

**Figure 3:**
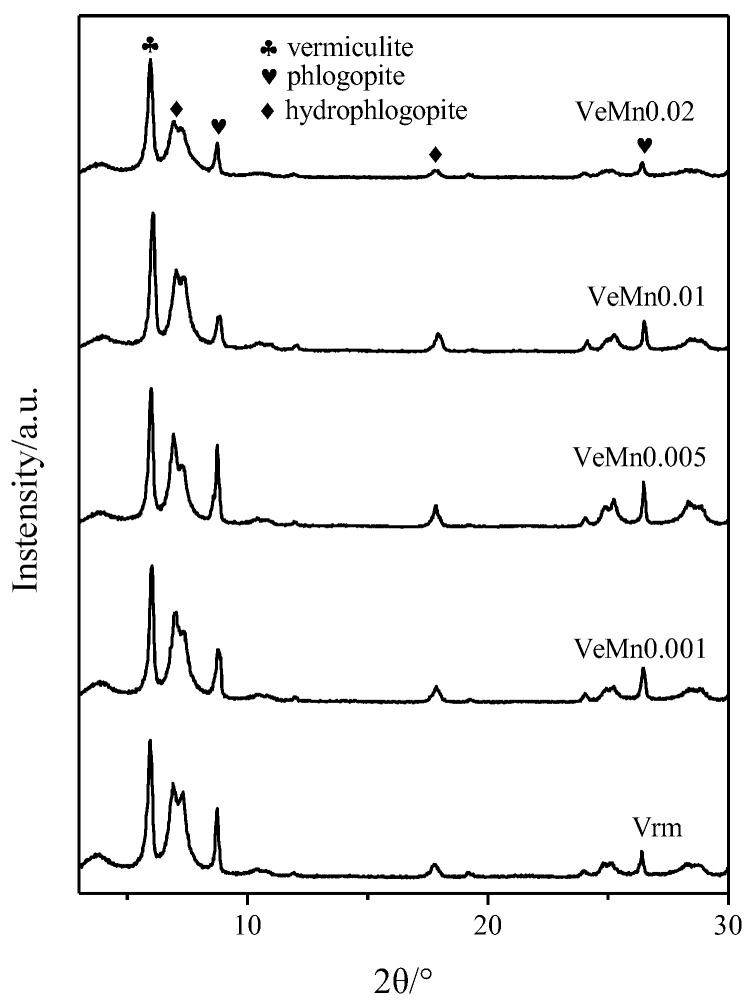
Effect of manganese dioxide on crystal structure.

**Figure 4:**
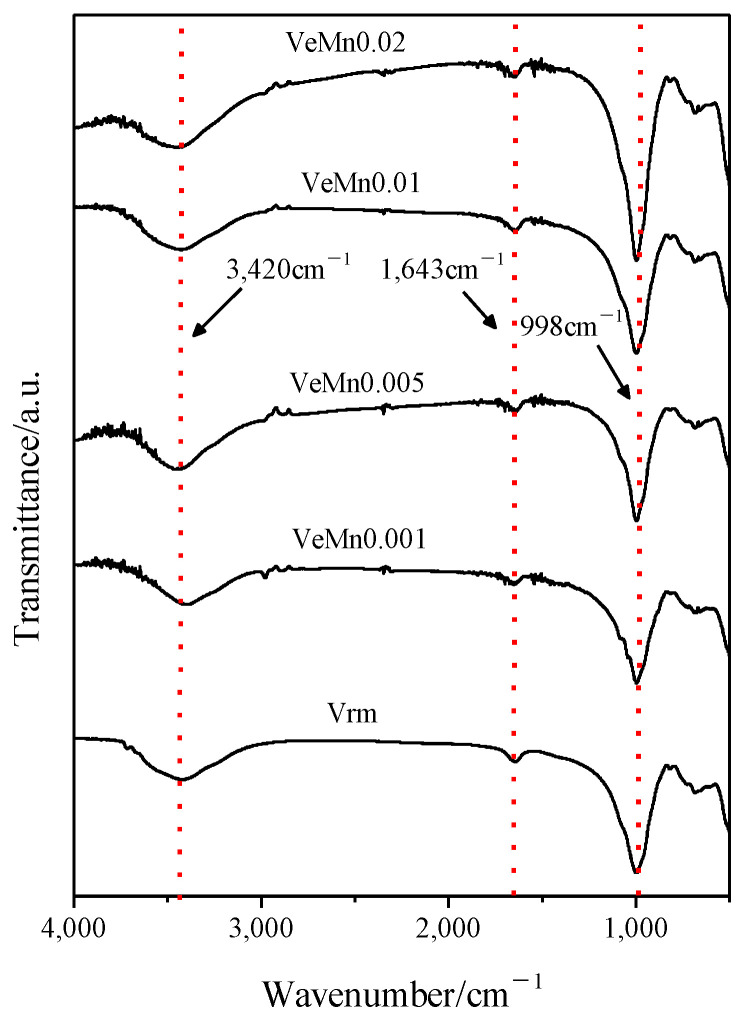
Effect of manganese dioxide on the structure of functional groups.

Then the morphology was analyzed. The results of XRD and Fourier transform infrared (FTIR) tests are shown in Figure 3 and Figure 4. It can be seen that, the addition of manganese dioxide did not have a significant effect on the vermiculite crystal structure. The number of Vrm layers, hydrargyrite layers, and water molecule layers of the samples remained 2, 1 and 0 [23]. By reviewing the literature, 998 cm^−1^ is the Si-O-Si stretching vibrational band and 3420 cm^−1^ and 1643 cm^−1^ are the vibrational absorption bands of the water molecules [24]. Manganese dioxide did not cause any change in the structure of the functional groups. It was verified that peroxide expanded vermiculite did not cause structural damage. This is of great importance for the treatment of pollutants in the environment [25,26,27].

**Figure 5:**
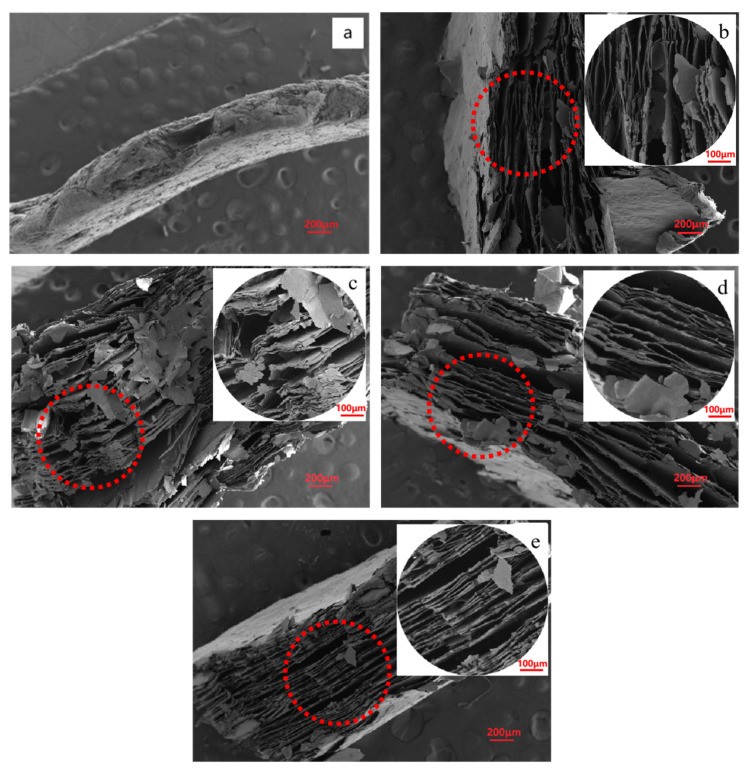
SEM images at different additions of manganese dioxide: (**a**) Vrm, (**b**) 0.001 g, (**c**) 0.005 g, (**d**) 0.01 g, and (**e**) 0.02 g.

To visualize the effect of manganese dioxide on the expansion, SEM tests were performed, as shown in Figure 5. Figure 5b–e shows the microstructure of the samples with different manganese dioxide. At 0.001 g, the Vrm layer was the thinnest and the average layer spacing was the largest. With the increase in MnO_2_, the interlayer spacing of Vrm gradually decreased. The thickness of the layer also increased. The Vrm layers were not exfoliated at 0.02 because too fast an exothermic rate cannot reach expansion. It is consistent with the results in Figure 1. That is, the expansion rate was the largest at 0.001 g of MnO_2_. It is calculated that the exothermic rate at this time was 1960.42~2089.164 J/min. At this rate, the expansion effect is the best. It can effectively increase the volume of Vrm and affect the specific surface area, which is favorable for the adsorption of pollutants [28]. Therefore, 0.001 g will be selected for the follow-up study.

### 2.2. Effect of Solid–Liquid Ratio

**Figure 6:**
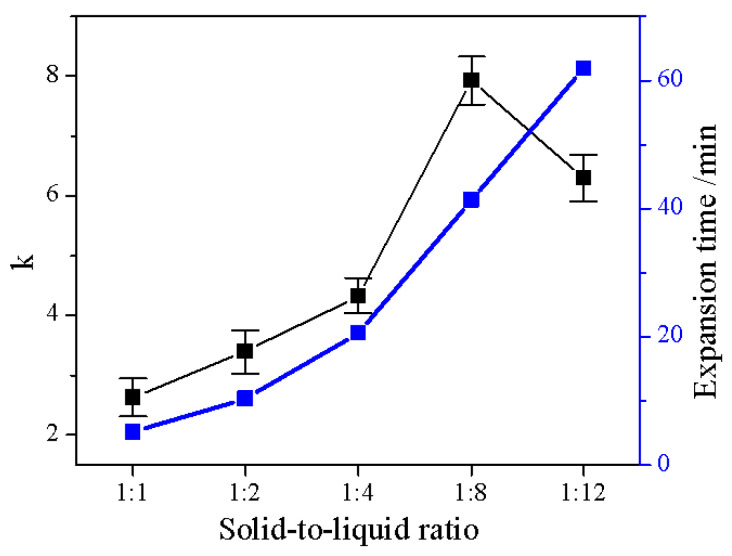
The effect of solid–liquid ratio on expansion rate and expansion time.

After the amount of manganese dioxide was determined, the ratio of vermiculite to hydrogen peroxide was also discussed. A suitable solid–liquid ratio can effectively reduce the waste of resources. Five different solid–liquid ratio conditions were designed. Figure 6 shows the variation of expansion time and k. Among all samples, the best performance was 1:8. Compared to 1:1, k was increased by about 3 times, k≈8. The expansion time was about 40 min. Marcos [13] found that vermiculite immersed in 30% hydrogen peroxide had a k of about 6 after 4 h. The expansion efficiency and k were significantly lower than VeH8. Combined with Figure 6, it can be seen that the variation of k can be divided into two periods. At the solid–liquid ratio greater than 1:8 (*w*/*v*), k tended to increase. When it is smaller than 1:8 (*w*/*v*), k fell back. The expansion time showed an increasing trend. It showed that the trend of expansion time and k was related to hydrogen peroxide, but k decreased after exceeding a certain amount. The amount of hydrogen peroxide determined the total heat release of the reaction. When the Vrm was 10 g, the total heat release of the five groups increased from 10,445.82 J to 125,349.84 J sequentially. At the same time, they had the same exothermic rate. Combined with the trend of k (Figure 6), it can be speculated that when the total amount of exotherm was too high, it was unfavorable for the expansion. To find out the effect of hydrogen peroxide on expansion, SEM, N_2_ adsorption–desorption tests will be performed on the samples later.

**Figure 7:**
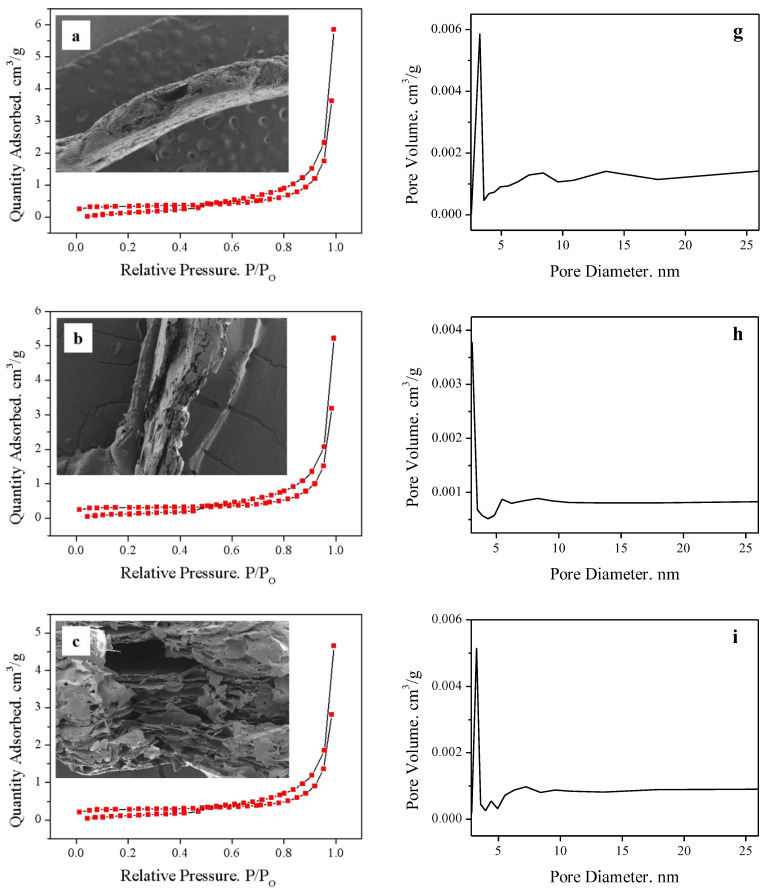

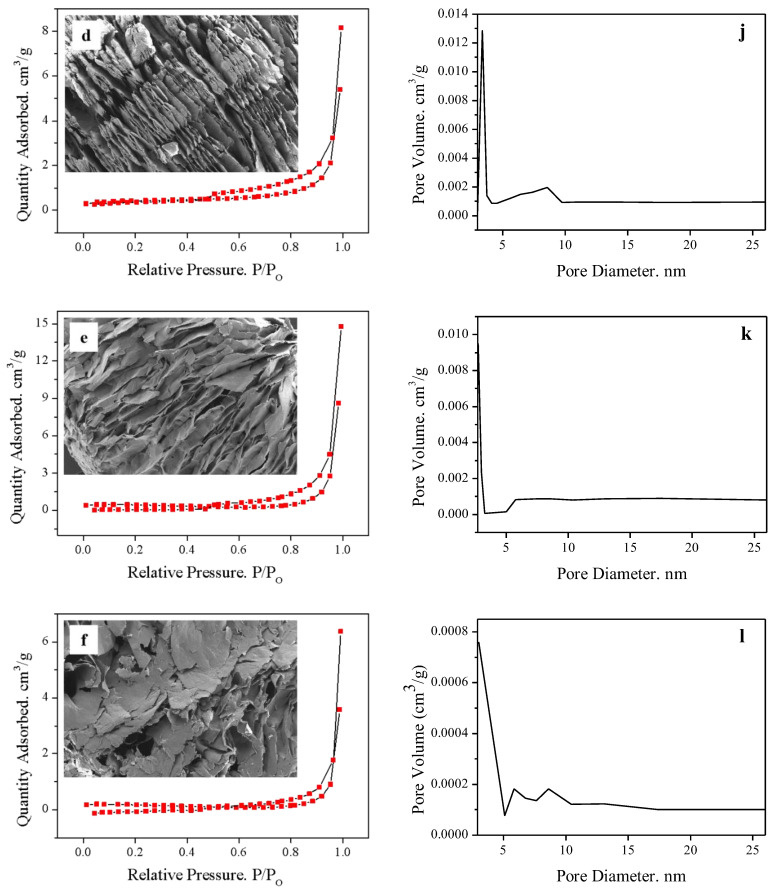
SEM images, N2 adsorption–desorption and pore diameter distribution curves for different solid–liquid ratios: 1:0 (**a**,**g**), 1:1 (**b**,**h**), 1:2 (**c**,**i**), 1:4 (**d**,**j**), 1:18 (**e**,**k**), 1:12 (**f**,**l**).

The SEM, N_2_ adsorption–desorption and pore diameter distribution results are shown in Figure 7 (N_2_ adsorption–desorption test was performed on the samples under 77 k liquid nitrogen conditions. The degassing conditions were 300 °C, 6 h). First, according to the pore diameter distribution curves, the pore size of all samples was mainly distributed around 3 nm, and no obvious trend was observed. Then the N_2_ adsorption–desorption curves were analyzed; it can be seen that all five groups have type V adsorption isotherm curves. At lower P/P0, the adsorbent material–adsorbent gas interactions were relatively weak. At higher relative pressures, an inflection point appeared, which indicates that the agglomerated molecules filled the pore channels. The hysteresis line in the high-pressure region was almost vertical and the adsorption–desorption curves are almost parallel there, which indicates that the pore size distribution of the sample was concentrated. The adsorption curves do not show the ultimate adsorption capacity in the high-pressure region, and the adsorption capacity increases monotonically with increasing pressure, indicating the presence of parallel plate or conical slit pores inside the sample [29]. The specific surface area of the sample was calculated to be in the range from 4 to 40 m^2^/g, with an increasing trend. However, it was still maximum at 1:8 and dropped back at 1:12. The highest N_2_ adsorption–desorption values were found in Figure 7e. It proves that N_2_ adsorption was maximum at 1:8. It coincided with the specific surface area results. Combined with the scan, the morphological changes were clearly seen. With the increase in hydrogen peroxide, the expansion effect became more and more obvious. In 1:8 (*w*/*v*) condition, the Vrm layer spacing was significantly larger than the other cases. The interlayer structure was propped open obviously, the sheet layer was smooth and complete, and the thickness was significantly smaller than the other samples. This may be the cause of the greatest amount of adsorption. However, at 1:12 (*w*/*v*) condition, the vermiculite lost its accordion-like interlayer structure and became disordered. Although more complete lamellae were seen, they adhered to each other and the pores were blocked. This may be the reason for the decrease in K value. It also explains why the N_2_ adsorption–desorption at 1:12 was less than 1:8 (*w*/*v*). With this conclusion, it can be seen that both the exothermic rate and the total exothermic heat have an effect on the expansion.

The decomposition of hydrogen peroxide is an advanced oxidation process in which hydroxyl radicals (HO^•^) are derived by the reaction of a metal ion (M^n+^) and hydrogen peroxide (H_2_O_2_) [30]. The reaction has been proposed to occur by the following mechanism [31]:

(1) $$M^{n+}+H_{2}O_{2}\to M^{\left(n+1\right)}+HO^{•}+HO^{-}\;$$

(2) $$H_{2}O_{2}+HO^{•}\to H_{2}O+HOO^{•}\;$$

(3) $$M^{\left(n+1\right)}+HOO^{•}\to M^{n+}+H^{+}+O_{2}\;$$

The hydrogen peroxide molecules penetrate between the vermiculite layers, decompose with the release of atomic oxygen, and the remaining protons combine with oxygen molecules to separate the silicate layers, leading to expansion [19].

The catalytic effect of manganese dioxide on hydrogen peroxide accelerates the decomposition reaction. The molecules entering the interlayer are also affected. Protons and oxygen molecules also combine quickly. This increases the expansion efficiency. The study indicates that the adsorption properties of Vrm are related to the degree of expansion [18]. It is important for the use of Vrm to adsorb pollutants from the environment. The results of this experiment show that when using hydrogen peroxide to expand Vrm, it is not the case that the greater the amount of hydrogen peroxide, the better the expansion effect. Too much hydrogen peroxide can lead to the destruction of the interlayer structure, making the original regular interlayer arrangement disordered. The finding is of great significance for the green development of the Vrm expansion process.

### 2.3. Adsorption Experiment

By exploring the optimal solid–liquid ratio, it was initially determined that the expanded Vrm obtained under the condition of solid–liquid ratio of 1:8 (*w*/*v*) when the amount of manganese dioxide was 0.001 g had a better expansion effect. Based on this conclusion, it was boldly assumed that the adsorption rate of VeH8 to pollutants in the environment was the largest among the five groups of samples under the same conditions. To confirm this hypothesis, a set of adsorption experiments will be designed.

**Figure 8:**
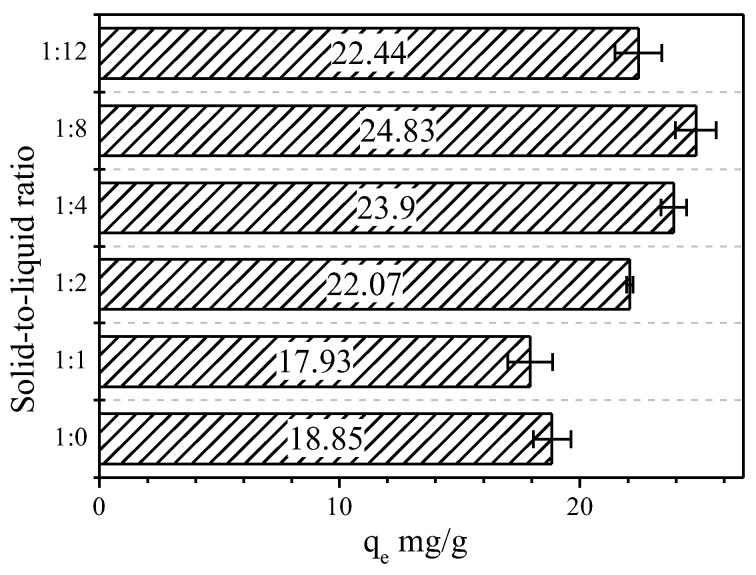
Effect of solid–liquid ratio on Cd adsorption.

The initial concentration of Cd ions was 100 mg/L, pH = 6 and a temperature of 297 K. All samples reached adsorption equilibrium after 180 min. By calculating the equilibrium adsorption amount (Figure 8), the trend of the adsorption amount of the samples is consistent with k in Figure 6. The trend of the adsorption amounts with hydrogen peroxide was basically the same. However, the adsorption amounts dropped back at 1:12. All experimental groups were higher than Vrm except VeH1. VeH8 increased by about 30% over Vrm. The above speculation was verified. In other words, the expansion is large and regular adsorption capacity is strong.

To investigate the mechanism of adsorption and possible rate control steps, kinetic models were used to fit experimental data. The pseudo-first order kinetics and pseudo-second order kinetics models were fitted, respectively. The expressions for the two different kinetic models are as follows (Equations (1) and (2)), where *q_e_*, *q_t_*, *k*_1_, *t*, and *k*_2_ represent the equilibrium adsorption amount, the adsorption amount of the sample at different times, the rate constant of the pseudo-first-order kinetic model, the adsorption time, and the rate constant of the pseudo-second-order kinetic model, respectively.

(4) $$\ln\left(q_{e}-q_{t}\right)=lnq_{e}+k_{1}t$$

(5) $$\frac{t}{q_{t}}=\frac{1}{k_{2}q_{e}^{2}}+\frac{t}{q_{e}}\;$$

**Figure 9:**
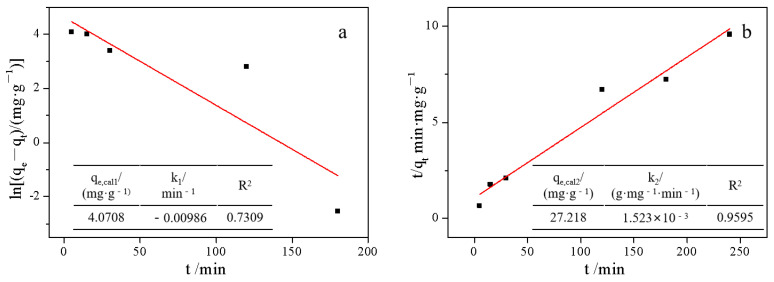
Pseudo-first-order kinetic (**a**) and pseudo-second-order kinetic (**b**) fitting curves for VeH8.

Figure 9 shows the linear fitting curves of the pseudo-first-order kinetics and pseudo-second-order kinetics of Cd adsorption by VeH8. From Figure 9, it can be seen that the pseudo-first order kinetics model was a poor overall fit to the kinetic experimental data of Cd adsorption by VeH8, R^2^ = 0.7309. The difference with the actual results is large. The pseudo-second order kinetics model fits well to the experimental data of adsorption kinetics, R^2^ = 0.9595. The maximum saturated adsorption capacity calculated as 27.218 mg/g (*q_e, cal2_*), which was similar to the experimental data. Therefore, the use of the pseudo-second order kinetics model can better reflect the process of Cd adsorption by VeH8. This is similar to the results of other adsorption studies of Vrm [32].

It was demonstrated that the adsorption rate of Cd by VeH8 was proportional to the square of the concentration of Cd. The metal ions were adsorbed on Vrm via several mechanisms, such as the cation exchange at the planar sites, the formation of inner-sphere complexes through the Si–O^−^ and Al–O^−^ groups at the clay particle edges, and mainly the introduction inside the lamellar space of this clay [8]. This indicates that the adsorption process of Cd on VeH8 was not only influenced by the external mass transfer resistance, but there were multiple adsorption mechanisms, including liquid film diffusion, internal diffusion, and chemisorption [33].

## 3. Materials and Methods

### 3.1. Materials

In this study, Vrm of Xinjiang Yuli was selected. With diameters of 2~4 mm and thicknesses of 0.3~0.5 mm. This Vrm has a 1:1 regular interlayer structure [34]. The chemical composition of Vrm by X-ray fluorescence (XRF) spectrometer is listed in Table 1. The bulk density of Vrm is 898.46 kg/m^3^.

### 3.2. Preparation of Samples

The concentration of hydrogen peroxide used in the experiment was 30% (Z ≥ 30.0% (volume, V_H_2_O_2__/V_H_2_O_); AR, Tianjin Guangfu fine chemical research institute, Tianjin, China). When discussing the amount of manganese dioxide (AR, Xilong scientific, Shantou, China) hydrogen peroxide and Vrm were 40 mL, 10 g. When discussing the solid–liquid ratio, Vrm was 10 g, and hydrogen peroxide was adjusted according to the specific situation. The specific scheme is shown in Table 2.

To exclude the effect of soaking time on expansion, all groups completed the addition of manganese dioxide within 5 min. The reaction was completed when no visible bubbles were produced. After finishing, the samples were rinsed 3–5 times with deionized water, then dried in an oven at 65 °C and stored in bags. The samples were crushed for 20 s and tested for expansion rate, specific surface area, and adsorption properties.

### 3.3. Cd adsorption Experiment

The static batch experiment method was used. The equilibrium adsorption of Cd was investigated at a concentration of 100 mg/L, a temperature of 297 K, and pH = 6. The adsorption process of the optimal group was fitted with the proposed primary kinetics and the proposed secondary kinetics. Three parallel samples were made under the same conditions, and the results were averaged.

Weigh 5 mg of the sample into a 100 ml centrifuge tube, add 50 ml of Cd solution and shake uniformly. After centrifugation at high speed (8000 r/min), the solid–liquid separation was carried out using membranes with a pore size of 0.45 μm, and the Cd concentration in the filtrate was determined.

The concentration of Cd solution was determined by ICP-OES. The equilibrium adsorption amount (*q_e_*) of the sample was calculated according to Equation (6), where *q_e_, C_0_, C_e_, V, m* represent the equilibrium adsorption amount, the initial concentration, the equilibrium adsorption concentration, the volume of Cd solution, and the sample mass.

(6) $$q_{e}=\frac{\left(C_{0}-C_{e}\right)V}{m}$$

### 3.4. Characterization

The chemical compositions of the samples were determined via X-ray fluorescence spectroscopy (XRF, Axios, PANalytical, Almelo, The Netherlands). The phase compositions of the samples were identified via X-ray diffraction (XRD, D/MAX-IIIB, Rigaku, Tokyo, Japan). The microscopic morphology of the samples was observed using by scanning electron microscopy (SEM/EDS, Ultra55, Zeiss, Jena, Germany). Functional group changes of the samples were recorded using a Fourier transform infrared spectrometer (FTIR, Magna 550II, Perkinlemer Frontier, Waltham, MA, USA). Surface area and N_2_ adsorption–desorption were measured with Brunauer–Emmett–Teller (BET, Micromeritics ASAP 3020, Atlanta, GA, USA). The Cd concentration in solution was analyzed by inductively coupled plasma emission spectrometry (ICP-OES, iCAP 5110 full spectrometer, Shanghai, China). The expansion rate k was measured by the change in the bulk density, k = $\frac{Experimental\;group\;bulk\;density\;}{\mathrm{Vrm}\;\mathrm{bulk}\;\mathrm{density}}$. The bulk density was measured by the method in the national standard JC/T 441-2009 [35] “Expanded Vrm”.

## 4. Conclusions

The swelling of Vrm by hydrogen peroxide under the catalytic effect of manganese dioxide did not change the interlayer structure and functional groups. With the increase in manganese dioxide, it led to an accelerated exothermic rate. Although the expansion time was greatly reduced, the expansion effect decreased. The study on the amount of manganese dioxide added showed that the exothermic rate was 1960.42–2089.164 J/min when 0.001 g. The expansion effect was best at this time.

Then the amount of hydrogen peroxide was studied. It was found that the heat required to achieve the ideal expansion was 39,208.4~41,783.28 J at 10 g Vrm. Too little heat would lead to incomplete expansion and too much heat would lead to the destruction of the interlayer structure of Vrm. Neither can achieve the ideal expansion, affecting k and the specific surface area. Eventually, the adsorption performance of the sample is affected.

The VeH8 prepared according to the above optimal data showed an improvement of about 30% in the adsorption of Cd compared to Vrm in accordance with the pseudo-second-order kinetic model. The method swells 10 g of Vrm in only 40 min, which is much more efficient. This is of great significance for improving the preparation efficiency of expanded Vrm. More importantly, the prepared samples can be used to treat heavy metals in the environment.

## Figures and Tables

**Table 1:** Distribution of various components (wt. %) of Vrm.

Component	SiO_2_	MgO	Al_2_O_3_	Fe_2_O_3_	K_2_O	Na_2_O	TiO_2_	CaO	F	BaO	LOI
Content	38.32	20.18	12.34	7.36	4.86	1.50	1.39	1.26	0.16	0.10	12.53
*LOI (loss on ignition)											

**Table 2:** Experimental scheme of expansion.

No.	Vrm: Hydrogen Peroxide (g:mL)	Manganese Dioxide Addition (g)
VeMn0.001	1∶4	0.001
VeMn0.005	1∶4	0.005
VeMn0.01	1∶4	0.01
VeMn0.02	1∶4	0.02
VeH1	1∶1	0.001
VeH2	1∶2	0.001
VeH4	1∶4	0.001
VeH8	1∶8	0.001
VeH12	1∶12	0.001

## Data Availability

Not applicable.
